# Carvedilol Decrease IL-1β and TNF-α, Inhibits MMP-2, MMP-9, COX-2, and RANKL Expression, and Up-Regulates OPG in a Rat Model of Periodontitis

**DOI:** 10.1371/journal.pone.0066391

**Published:** 2013-07-03

**Authors:** Raimundo Fernandes de Araújo Júnior, Tatiana Oliveira Souza, Caroline Addison Xavier de Medeiros, Lélia Batista de Souza, Maria de Lourdes Freitas, Hévio Freitas de Lucena, Maria do Socorro Costa Feitosa Alves, Aurigena Antunes de Araújo

**Affiliations:** 1 Post Graduation Program in Functional and Structural Biology**/**Post Graduation Program Health Science/Department of Morphology, UFRN, Natal, Rio Grande do Norte, Brazil; 2 Post Graduation Program Health Science/UFRN and FACENE, Mossoró, Rio Grande do Norte, Brazil; 3 Post Graduation Program in Health and Society/UERN, Mossoró, Rio Grande do Norte, Brazil; 4 Postgraduation Program Oral Pathology/UFRN, Natal, Rio Grande do Norte, Brazil; 5 Department of Morphology, UFRN, Natal, Rio Grande do Norte, Brazil; 6 Department of Dentristry/UFRN, Natal, Rio Grande do Norte, Brazil; 7 Postgraduation Program Health Science/Postgraduation Program Public Health/UFRN, Natal, Rio Grande do Norte, Brazil; 8 Postgraduation Program Public Health/Postgraduation Program in Pharmaceutical Science/Department of Biophysics and Pharmacology, UFRN, Natal, Rio Grande do Norte, Brazil; National Institutes of Health, United States of America

## Abstract

Periodontal diseases are initiated primarily by Gram-negative, tooth-associated microbial biofilms that elicit a host response that causes osseous and soft tissue destruction. Carvedilol is a β-blocker used as a multifunctional neurohormonal antagonist that has been shown to act not only as an anti-oxidant but also as an anti-inflammatory drug. This study evaluated whether Carvedilol exerted a protective role against ligature-induced periodontitis in a rat model and defined how Carvedilol affected metalloproteinases and RANKL/RANK/OPG expression in the context of bone remodeling. Rats were randomly divided into 5 groups (n = 10/group): (1) non-ligated (NL), (2) ligature-only (LO), and (3) ligature plus Carvedilol (1, 5 or 10 mg/kg daily for 10 days). Periodontal tissue was analyzed for histopathlogy and using immunohistochemical analysis characterized the expression profiles of MMP-2, MMP-9, COX-2, and RANKL/RANK/OPG and determined the presence of IL-1β, IL-10 and TNF-α, myeloperoxidase (MPO), malonaldehyde (MDA) and, glutathione (GSH). MPO activity in the group with periodontal disease was significantly increased compared to the control group (p<0.05). Rats treated with 10 mg/kg Carvedilol presented with significantly reduced MPO and MDA concentrations (p<0.05) in addition to presenting with reduced levels of the pro-inflammatory cytokines IL-1 β and TNF-α (p<0.05). IL-10 levels in Carvedilol-treated rats remained unaltered. Immunohistochemical analysis demonstrated reduced expression of MMP-2, MMP-9, RANK, RANKL, COX-2, and OPG in rats treated with 10 mg/kg Carvedilol. This study demonstrated that Carvedilol affected bone formation/destruction and anti-inflammatory activity in a rat model of periodontitis.

## Introduction

It is well established that ß-blockers substantially improve symptoms and the outcome of patients presenting with chronic heart failure (CHF) and left ventricular systolic dysfunction [1]. Carvedilol is a non-selective β-blocker with alpha (1)-adrenergic receptor antagonistic properties. It is unique among ß-blockers because (in addition to improving exercise tolerance and its anti-ischemic properties) it reduces heart rate and myocardial contractility [2].

Carvedilol exerts antioxidant [3], [4] and anti-inflammatory [3], [5] effects. The antioxidant effects of Caverdilol were determined following the identification of lipid peroxidation by-products such as malondialdehyde [6]–[8] and glutathione [9], [10] and its anti-inflammatory properties following the observation that Caverdilol reduced pro-inflammatory cytokine production combined with increased anti-inflammatory cytokine (e.g., IL-10) production [5]. Another interesting finding was the observation that diabetic animals treated with Caverdilol had reduced diabetes associated low-turnover bone disease beyond what can be attributed to its antioxidative stress mechanism [11].

Examination of the effects of caverdilol in periodontal disease is of interest for chronic heart failure patients since induction of local inflammatory process (e.g., as a consequence of bacterial infections seen in periodontal disease) may aggravate their heart condition since correlations between periodontal disease and atherosclerosis [12], [13] have been established.

Dysregulation of myocardial metalloproteinases (MMPs) is now regarded as an early contributing factor to the initiation and progression of heart failure and pre-treatment with Carvedilol prevented MMP-2 and MMP-9 expression [14] shown to be activated in periodontal disease [15].

The aim of present study was to determine the efficacy of Carvedilol in the treatment of periodontal disease by assessing its anti-inflammatory and antioxidant properties while also characterizing the expression profile of matrix metalloproteinases and bone markers during treatment.

## Materials and Methods

### Animals

Experiments were performed using male wistar rats (180–220 g) housed in standard conditions (12 h light/dark cycle at 22±0.1°C). Animals had free access with *ad libitum* access to water and standard diet (Presence/Evialis do Brasil Nutrição Animal LTDA, São Paulo ). The experimental protocol was approved by the Animal Ethics Committee (number 28/2012) of the Federal University of Rio Grande Norte, Brazil.

### Drug Treatments

Carvedilol (Cardilol, LIBBS, São Paulo, Brazil) was solubilized in saline (vehicle). All treatments (carvedilol or vehicle) were given orally by gavage 1 h before ligation (induction of EPD) and thereafter once daily for 10 days. The animals were assigned randomly to the following five groups, with 10 animals for group: (1) a non-ligated group that received saline (NL), (2) a ligated group that received saline (L), (3) a ligated group treated with 1 mg/kg carvedilol (1 mg/kg CARVE), (4) a ligated group a group treated with 5 mg/kg carvedilol (5 mg/kg CARVE), and (5) a ligated group a group treated with 10 mg/kg carvedilol (10 mg/kg CARVE). On the 11^th^ treatment day animals were euthanized using thiopental (20 mg/kg).

### Induction of Experimental Periodontitis (EPD)

Experimental periodontitis was induced under ketamine (Quetamina, VETNIL 10%, São Paulo, 70 mg/kg, i.p) and xylazine anesthesia (Calmium 2%, São Paulo, 10 mg/Kg, i.p) and then placing a sterile nylon (3-0 Polysuture, NP45330, São Paulo) thread ligature around the cervix of the left second maxillary molar. At the end of the experiment animals were euthanized using thiopental (Thiopentax 0.5 g, Cristália, São Paulo, 20 mg/kg).

### Histopathological Analysis

The immunohistochemical analysis and the histological scores of the periodontal tissues were conducted by two calibrated oral pathologists. The sectioning was performed in the laboratoryof Morphology and Oral Pathology and subsequently analyzed by light microscopy in the Department of Morphology, UFRN. Maxillae from animals in the respective treatment groups were excised following euthanasia. Specimens were fixed in 10% neutral buffered formalin and demineralized in 5% nitric acid. Specimens were then dehydrated, embedded in paraffin, and sectioned (4 µm thickness) along the molars in a mesio-distal plane prior to hematoxylin and eosin (H&E) staining. Sections corresponding to the area between the first and second molars where a ligature had been placed were evaluated by light microscopy (40X magnification). Parameters including the degree of inflammatory cell influx and alveolar bone and cementum integrity were analyzed histologically in a single-blind fashion and graded as follows: 0, absence of or only discrete cellular infiltration (inflammatory cell infiltration is sparse and restricted to the region of the marginal gingiva), preserved alveolar process and cementum; 1, moderate cellular infiltration (inflammatory cellular infiltration present all over the gingiva), some but minor alveolar process resorption and intact cementum; 2, accentuated cellular infiltration (inflammatory cellular infiltration present in both the gingival and periodontal ligament), accentuated degradation of the alveolar process, and partial destruction of cementum and 3, accentuated cellular infiltrate, complete resorption of the alveolar process and severe destruction of the cementum [16].

### Immunohistochemistry: COX-2, MMP-2, MMP-9, RANK-L, RANK, and OPG Detection

Periodontal tissue sections (4 µm) were transferred onto gelatin-coated slides, deparaffinized, and then rehydrated. Gingival and periodontal tissue slices were then washed with 0.3% Triton X-100 in phosphate buffered saline (PBS) and endogenous peroxidase quenched following an incubation with 3% hydrogen peroxide. Sections were then were incubated with primary antibodies (Santa Cruz Biotecnology) at a 1∶400 dilution specific to either cyclooxygenase-2 (COX-2), metalloproteinase-2 (MMP-2), metalloproteinase-9 (MMP-9), receptor activator of the nuclear factor-kB ligand (RANK-L), receptor activator of the nuclear factor-kB (RANK), osteoprotegerin (OPG) overnight at 4°C. After washing with PBS, slices were then incubated with the secondary antibody (Biocare Medical) for 30 min and immunoreactivity to COX-2, MMP-2, MMP-9, RANK, RANK-L, and OPG visualized using a colorimetric-based detection kit following the manufacturer’s protocol (TreekAvidin-HRP Label+Biocare Medical Kit, DAKO, USA).

### Myeloperoxidase Assay

The extent of neutrophil accumulation in respective gingival samples was measured by assaying myeloperoxidase (MPO) activity. Gingival samples were removed and stored at −70°C until used. After homogenization and centrifugation (2000×g for 20 min) MPO activity was determined by a colorimetric method described previously [17]. The results were reported as units of MPO/mg of tissue.

### Malonaldehyde Levels

Lipid peroxidation in rat gingival tissue was determined by measuring malonaldehyde (MDA) production via the thiobarbiturc reaction. Briefly, 0.25 ml of a 10% gingival tissue homogenate prepared in 0.15 M KCl was added to 1.5 ml of 1% H_3_PO_4_ and 500 µl of 0.6% thiobarbituric aqueous solution and then placed in a water bath for 45 min at 100°C and 2 ml of n-butanol P.A. was added before the mixture was homogenized and then centrifuged at 12000 rpm fro 15 min at 4°C. The absorbance of the butanol layer was measured at 520 (A1) and 535 nm (A2). The concentration of malonaldehyde was the difference between the A2– A1 values expressed as nM of MDA/g of gingival tissue.

### Glutathione Assay

Glutathione levels were determined as a measure of antioxidant activity. Gingival samples were removed and stored at −70°C until used. Briefly, 0.25 ml of a 5% gingival tissue homogenate resuspended in 0.02 M EDTA was added to 320 µl of distilled water and 80 µl 50% TCA and then centrifuged at 3000 RPM for 15 min at 4°C. 400 µl of the supernatant was then added to 800 µl 0.4 M TRIS buffer, pH 8.9, and 20 µl 0.01 M DTNB. Glutathione levels were determined by a colorimetric method described previously (17). The results were reported as units of MPO/mg of tissue and the absorbance measured at 420 nm.

#### IL-1β, IL-10, and TNF-α assay

The gingival sample tissues were stored at −70°C until required for each assay. The tissue collected was homogenized and processed as described by [18]. The levels of IL-1β, Il-10, and TNF-α in the gingival samples were determined with an ELISA commercial kit (R&D Systems, EUA) as described previously [19]. Briefly, micro titer plates were coated overnight at 4°C with antibodies against mouse TNF-α, IL-1β, and Il-10. After the plates were blocked, the samples and standards were added at various dilutions in duplicate and incubated at 4°C for 24 h. The plates were washed three times with buffer. The following antibodies were then added to the wells:biotinylated sheep polyclonal anti-TNF-α,anti-IL-1β, or anti-IL-10 (diluted 1∶1000 with 1% BSA assay buffer). After further incubation at room temperature for 1 h, the plates were washed, and 50 µl of avidin-HRP (diluted 1∶5000) was added. The color reagent o-phenylenediamine (OPD; 50 µl) was added 15 min later, and the plates were incubated in the dark at 37°C for 15–20 min. The enzyme reaction was stopped with H_2_SO_4_,and absorbance was measured at 490 nm. The resulting values were expressed in pg/ml.

#### Statistical analysis

The data are presented as means+standard error of the mean (SEM) or as medians, where appropriate. Analysis of Variance (ANOVA) followed by Bonferroni’s test was used to calculate the means, and the Kruskal–Wallis test followed by Dunn’s test was used to compare medians (GraphPad PRISM 5.0 Software). A *P*-value of <0.05 was considered to indicate a significant difference.

## Results

Animals treated with Caverdilol 10 mg/kg had significantly less alveolar bone loss and periodontal disease than rats treated with Caverdilol 1 mg/kg (p<0.05). In addition, rats in the 10 mg/kg group presented with gingival tissues containing discrete cellular infiltrates (restricted to the marginal gingival region) with preserved alveolar process and cementum.

**Figure 1:**
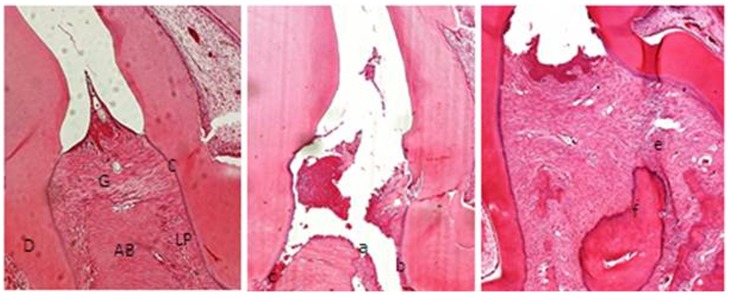
Microscopic analysis. **(A) Normal periodontium and (B) periodontium from a rat presenting with periodontitis (treated with saline) showing alveolar bone and cementum resorption and inflammatory cell infiltration.** (C) Reduced inflammation and alveolar bone loss in the periodontium of rats treated with Carvedilol (10 mg/kg) for 10 days. Sections were stained with H&E. Microscopic original magnification at 40X. Scale bars = 100 µm. G = gingiva; PL = Periodontal ligament; D = dentin; AB = alveolar bone; C = Cementum; a = bone loss; b = resorption of cementum; c = inflammatory process; e, f = decreased inflammation process and bone loss.

Histological analysis of the region between the first and second molars of sham operated rats demonstrated tissues presenting with a normal periodontium where the gingiva (g), periodontal ligament (pl), alveolar bone (ab), and cementum (c) are clearly defined (Figure 1). Histopathological analysis of the periodontium of the animals subjected to experimental periodontitis that received no treatment (EPD) revealed inflammatory cell infiltrates coupled with severe cementum and alveolar process destruction (histopathology score of 3; Figure 1B and Table 1). Carvedilol (10 mg/kg) treatment prevented inflammation induced by experimental periodontitis (Figures 1C) with rats in this group receiving a median histopathologic score of 1–2 (Table 1).

**Table 1:** Histological analysis of maxillae from rats presenting with periodontal disease, Natal, RN, 2013.

NL	L	CARVE 1 mg/kg	CARVE 5 mg/kg	CARVE 10 mg/kg
0 (0–0)	3 (3–3)*	3 (3–3)*	2 (1–2)	1 (1–2)

* p<0.05.

### Immunohistochemical Detection of COX-2, MMP-2, MMP-9, RANK-L, RANK, and OPG

**Figure 2:**
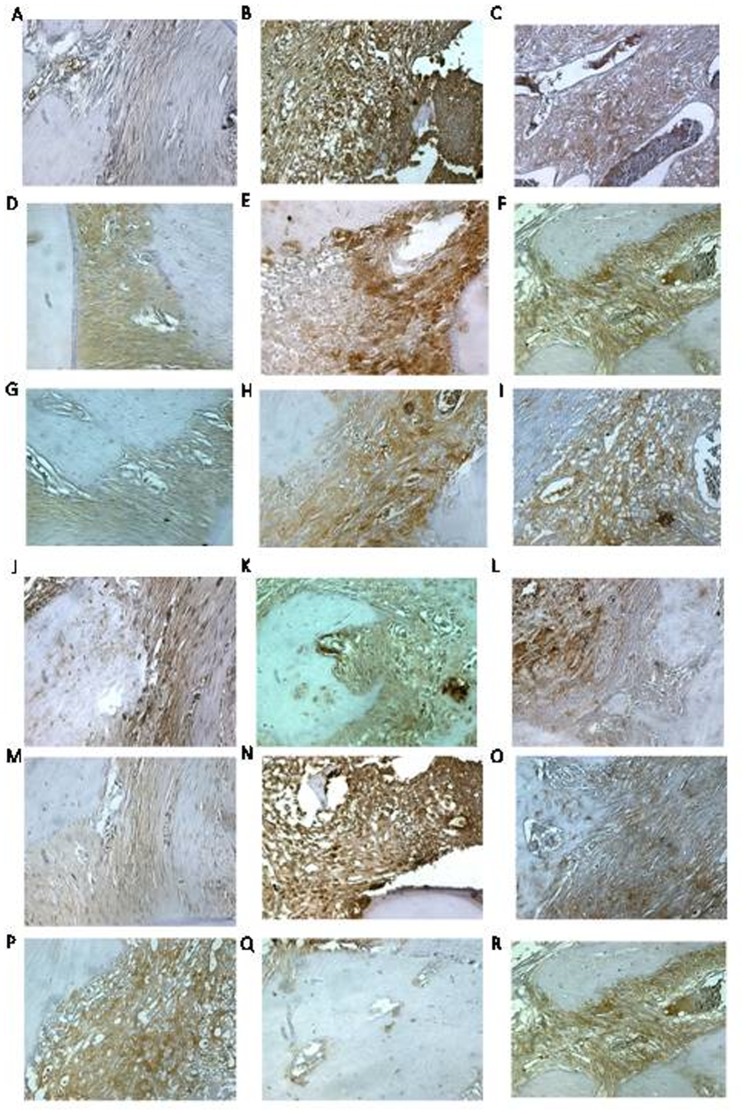
Immunoreactivity to MMP-2, MMP-9, COX-2, RANK, RANK-L, and OPG. Photomicrographs of periodontal tissues from rats presenting with EPD and treated with Carvedilol (10 mg/kg) (A, D, G, J, M, P). Rats treated with saline only (B, E, H, K, N, Q) and untreated rats presenting with EPD (C, F, I, L, O, R). Magnification 40X, bar scale = 100 µm. Pulp tissue (P), gingiva (G), periodontal ligament (PL), and dentina (D).

The periodontium of rats presenting with experimental periodontitis receiving no treatment (EPD) showed marked immune-staining for to the following markers: MMP-2, MMP-9, COX-2, RANK-L, and RANK (Figure 2B, E, H, K, and N) compared to periodontium staining profile of the saline group (Figure 2A, D, G, J, and M). Carvedilol (10 mg/kg) treatment reduced the levels of COX-2, MMP-2, MMP-9, RANK-L, and RANK expression in the periodontium of rats subjected to experimental periodontitis (Figure 2C, F, I, L, and O). By contrast, OPG staining was slightly elevated in the periodontal disease group, moderatly elevated in the saline group, and significantly up-regulated in the Carvedilol (10 mg/kg) group.

### Reduction of the Inflammatory Response

**Figure 3:**
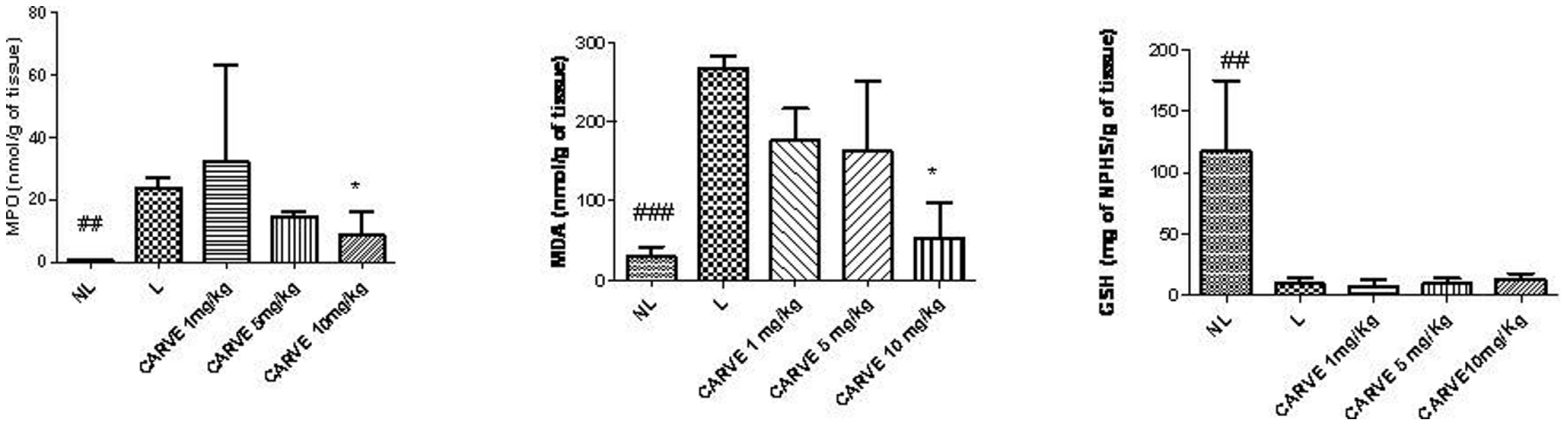
MPO, MDA, and GSH levels were measured in the NL, L and CARVE 1, 5, and 10 mg/kg groups. #,*p<0.05.

**Figure 4:**
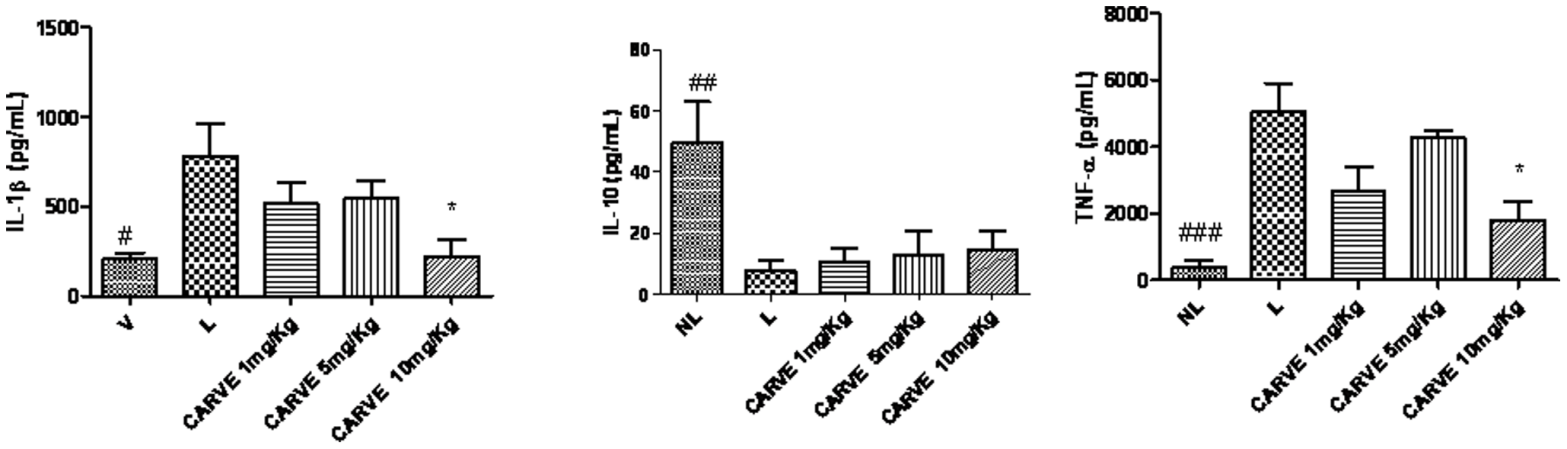
IL-1β, IL-10, and TNF-α levels were measured in the NL, L and CARVE 1, 5, and 10 mg/kg groups. ^#,*^p<0.05;

The MPO activity in rats presenting with periodontal disease was significantly increased compared to control animals (p<0.001). By contrast, rats treated with Carvedilol (10 mg/kg) had significantly reduced MPO concentrations (p<0.05, Figure 3). The levels of the proinflammatory cytokines IL-1β and TNF-α were significantly decreased in the Carvedilol (10 mg/kg; p<0.05) treatment group. IL-10 levels between treatment groups were not different (Figure 4).

### Reduction of Oxidative Stress

Treatment with Carvedilol (10 mg/kg) significantly reduced MPO activity (p<0.05), however, GSH levels were unaltered when compared to GSH levels in rats presenting with periodontal disease and treated with vehicle (Figure 3).

## Discussion

Neutrophils represent key phagocytic defenders in the periodontal pocket. Essentially, the stronger the inflammatory stimulus, the greater the epithelial and endothelial activation and the larger the number of neutrophils recruited. Recent evidence has shown that neutrophil-derived proteases also modulated chemokine activity [20] and that these proteases activated production of some cytokines including TNF-α and IL-1β [21]. Multiple neutrophil-derived proteolytic enzymes have been shown to be elevated in periodontitis compared to healthy controls, including myeloperoxidase (GCF) [22] and matrix metalloproteinases (e.g., MMP-2 and MMP-9) whose major source in the periodontium is the neutrophil [23]–[25].

This study confirmed that treatment with Carvedilol (10 mg/kg) reduced the levels of myeloperoxidase and malonaldehyde, in addition to reducing the levels of the the pro-inflammatory cytokines TNF-α and IL-1β. These observations are supported by previous studies that demonstrated that Carvedilol reduced cardiac gene expression and protein production of TNF-α, IL-1β, IL-6, and TGF-β_1_ in rats presenting with acute myocardial infarction [5].

Reduction in the inflammatory response can be confirmed by observing reduction in tissue COX-2 levels, an enzyme expressed in inflamed tissues, that was markedly reduced in rats treated with Carvedilol (10 mg/kg) compared to levels observed in rats presenting with periodontal disease.

Proteolytic enzymes released by host cells are associated with tissue destruction in periodontal diseases, specifically MMP-2 and MMP-9 have been implicated in periodontal disease progression. Histologic analysis demonstrated that rats treated with Carvedilol (10 mg/kg) had a decrease in staining for both MMP-2 and MMP-9, confirming previous reports that demonstrated that carvedilol reduced MMP-2 and MMP-9 levels [26]. Our study is innovative because it demonstrated a reduction in the formation of MMP-2 and MMP-9 in an experimental model of periodontal disease.

RANKL and OPG are members of the tumor necrosis factor (TNF) and TNF receptor (TNFr) super families, respectively, and binding to the receptor activator of NF-kB (RANK) not only regulates osteoclast formation (by mediating activation and survival in normal bone modeling and remodeling) but also regulates several other pathologic conditions characterized by increased bone turnover (26).

RANKL is synthesized in a membranous or soluble form by immune cells of the osteoblastic lineage cells. This factor binds the osteoclast surface receptor (RANK), stimulating bone resorption through osteoclastogenesis and the activation of multinucleated mature osteoclasts. OPG is secreted by osteoblasts as a decoy receptor for RANKL and prevents RANKL from binding to RANK thereby preventing bone resorption (27).

Recently, various basic and clinical research studies focusing on defining the underlying mechanisms of the major enzymatic drivers of this aggressive tissue destruction have been carried out. In addition to briefly discussing the pathology of chronic periodontitis and its main players, this article focused on describing promising therapeutic agents that can be used in the prevention of tissue destruction associated with periodontitis; i.e., using matrix metalloproteinase (MMP) inhibitors as host modulatory agents and bisphosphonates to block alveolar bone destruction.

Treatment with Carvedilol 10 (mg/kg) demonstrated its potential in reducing expression or RANK and RANKL in bone while increasin OPG expression resulting in a reduction in osteoclast activation and differentiation resulting in reduced bone loss. To our knowledge, no work has previoulsy demonstrted the activity of Carvedilol regarding the expression levels of either RANKL, RANKL, or OPG. This work demonstrated a new role for Carvedilol regarding signaling pathways involved in the process of bone formation/destruction suggesting that future work desinged to elucidate the mechanism involved in this process be carried out.

### After Discussion Conclusions

In conclusion, administration of Carvedilol 10 (mg/kg) confered anti-inflammatory activity by reducing the levels of myeloperoxidase, COX-2, IL-1β, and TNF-α. Carvedilol antioxidant activity was assessed by measuring malonaldehyde levels that post treatment were reduced. In summary, Carvedilol affected bone formation/destruction by reducing the levels of RANK and RANKL and increasing OPG expression.
